# Lichen Secondary Metabolites Inhibit the Wnt/β-Catenin Pathway in Glioblastoma Cells and Improve the Anticancer Effects of Temozolomide

**DOI:** 10.3390/cells11071084

**Published:** 2022-03-23

**Authors:** Aleksandra Majchrzak-Celińska, Robert Kleszcz, Elżbieta Studzińska-Sroka, Agnieszka Łukaszyk, Anna Szoszkiewicz, Ewelina Stelcer, Karol Jopek, Marcin Rucinski, Judyta Cielecka-Piontek, Violetta Krajka-Kuźniak

**Affiliations:** 1 Department of Pharmaceutical Biochemistry, Poznan University of Medical Sciences, Święcicki 4 Str., 60-781 Poznań, Poland; kleszcz@ump.edu.pl (R.K.); agnieszka.trzcinska0911@gmail.com (A.Ł.); anszoszk@gmail.com (A.S.); vkrajka@ump.edu.pl (V.K.-K.); 2 Department of Pharmacognosy, Poznan University of Medical Sciences, Rokietnicka 3 Str., 60-806 Poznań, Poland; elastudzinska@ump.edu.pl (E.S.-S.); jpiontek@ump.edu.pl (J.C.-P.); 3 Department of Histology and Embryology, Poznan University of Medical Sciences, Święcicki 6 Str., 60-781 Poznań, Poland; ewelina.stelcer@ump.edu.pl (E.S.); kjopek@ump.edu.pl (K.J.); marcinruc@ump.edu.pl (M.R.)

**Keywords:** lichen secondary metabolites, glioblastoma, Wnt/β-catenin pathway, caperatic acid, physodic acid, temozolomide, blood-brain barrier permeability, microarrays

## Abstract

Lichens are a source of secondary metabolites with significant pharmacological potential. Data regarding their possible application in glioblastoma (GBM) treatment are, however, scarce. The study aimed at analyzing the mechanism of action of six lichen secondary metabolites: atranorin, caperatic acid, physodic acid, squamatic acid, salazinic acid, and lecanoric acid using two- and three-dimensional GBM cell line models. The parallel artificial membrane permeation assay was used to predict the blood-brain barrier penetration ability of the tested compounds. Their cytotoxicity was analyzed using the MTT test on A-172, T98G, and U-138 MG cells. Flow cytometry was applied to the analysis of oxidative stress, cell cycle distribution, and apoptosis, whereas qPCR and microarrays detected the induced transcriptomic changes. Our data confirm the ability of lichen secondary metabolites to cross the blood-brain barrier and exert cytotoxicity against GBM cells. Moreover, the compounds generated oxidative stress, interfered with the cell cycle, and induced apoptosis in T98G cells. They also inhibited the Wnt/β-catenin pathway, and this effect was even stronger in case of a co-treatment with temozolomide. Transcriptomic changes in cancer related genes induced by caperatic acid and temozolomide were the most pronounced. Lichen secondary metabolites, caperatic acid in particular, should be further analyzed as potential anti-GBM agents.

## 1. Introduction

Lichens are a source of ~1000 unique secondary metabolites of various chemical structures, including aliphatic, cycloaliphatic, aromatic, and terpenic compounds [1,2]. These substances are usually deposited on the surface of mycelium cells and are mostly water-insoluble [3]. Their exact role in lichens is still debated, but it is suggested that they play an important role in lichen self-protection, including antioxidant, photoprotection, anti-microbial, and herbivore resistance [3]. They also contribute to metal and pollution resistance, as well as niche competition [3]. Although lichens have been used for medical purposes since ancient times, their full biopharmaceutical potential has only recently been discovered and appreciated. Lichen secondary metabolites, also called “lichenochemicals” exhibit a multitude of bioactivities, such as antioxidant, antibiotic, antiviral, anti-inflammatory, and others [4]. Recently, their anticancer properties have been intensively studied [2]. It has been shown that lichen-derived phenolic compounds, including anthraquinones, xanthones, dibenzofuranes, depsides, and depsidones can interfere with critical cancer-related signaling pathways and exert cytotoxic effects against cancer cells. The growing amount of data suggests that lichen secondary metabolites can enhance the anticancer effects of chemo- and radiotherapy, allowing the reduction of doses and ameliorating the side effects of currently used drugs [2]. Thus, they may provide new options for the development and research of adjuvant therapies or may even be regarded as the prototypes of novel anticancer drugs [5].

Glioblastoma (GBM) is the most common and devastating primary malignant brain tumor in adults. It has the astrocytic lineage and is classified as a WHO grade IV tumor [6]. With a 2 year survival rate of 26–33% and overall survival of only around 15 months, it is still one of the most challenging diseases of neuro-oncology [7]. Current standards of care of GBM patients include maximal tumor resection, followed by radiotherapy plus concomitant and maintenance chemotherapy with temozolomide (TMZ) [7]. Sadly, the survival rates for patients with GBM have shown no notable improvement in population statistics in the last three decades [6]. The inhibition of critical cancer-related signaling pathways and induction of apoptosis might offer the best opportunity to improve GBM patients’ prognosis.

Wnt/β-catenin pathway hyperactivation is one of the most important contributors to the malignant phenotype of GBM cells [8,9,10]. Physiologically, it regulates the critical cellular events during central nervous system development, including the self-renewal, differentiation, migration, and signaling of the neural stem cells in the fetal brain [11]. When cells such as stem/progenitor cells in the adult brain are not exposed to Wnt ligands, cytoplasmic β-catenin, a key molecule of the pathway, associates with a multi-protein “destruction complex”. This complex consists of adenomatous polyposis coli (APC), the axis inhibition proteins 1 and/or 2 (AXIN1/2), casein kinase 1 (CK1) and glycogen synthase kinase 3β (GSK3β) [11]. Phosphorylation of β-catenin by CK1 and GSK3β primes it for proteasomal ubiquitination. Thus, β-catenin levels are kept low. Without β-catenin, the T-cell factor/lymphoid enhancer factor (TCF/LEF) bound to Wnt responsive DNA elements (WREs) recruits transcriptional corepressor complexes. As a result, Wnt/β-catenin target gene expression is being repressed. However, in the abundance of Wnt ligands, the level of β-catenin increases. It is translocated to the nucleus, where together with TCF/LEF it stimulates the expression of target genes, including those responsible for the maintenance of stem cell properties [8]. The hyperactivated Wnt/β-catenin pathway is therefore related to the uncontrolled tumor growth, but also chemo- and radioresistance and worse prognosis [12]. Conversely, Wnt/β-catenin inhibition reduces the proliferation, survival, and clonogenicity of GBM cells [13].

In our previous studies, we discovered that lichen-derived extracts as well as lichen secondary metabolites, such as physodic acid (isolated from *Hypogymnia physodes*), evernic acid (isolated from *Evernia prunastri*), salazinic acid (derived from *Parmelia sulcata*), and (−)-usinc acid (from *Cladonia uncialis*) possess the anti-cancer properties and decrease the viability of GBM cells [5,14]. We also showed that physodic acid, evernic acid, and (−)-usnic acid can cross the blood-brain barrier (BBB), which makes them good prototypes of pharmacologically active compounds within the CNS, potentially suitable for the treatment of GBM [5,14]. *C. uncialis* is also the source of squamatic acid, which has not been analyzed in the context of GBM so far. The data about atranorin and caperatic acid, both derived from *Platismatia glauca*, and lecanoric acid from *Hypocenomyce scalaris*, in regard to GBM treatment, are also scarce.

Given this context, the main purpose of this study was to decipher the mechanism of action of lichen secondary metabolites, in particular in regard to Wnt/β-catenin pathway, and to verify if they could ameliorate the response of GBM cells to TMZ. Thus, the BBB permeability and the cytotoxicity of six lichen secondary metabolites, namely atranorin, caperatic acid, physodic acid, squamatic acid, salazinic acid, and lecanoric acid were evaluated using three GBM cell lines with different TMZ sensitivities. Their impact on the Wnt/β-catenin pathway was analyzed in the context of a single treatment as well as the co-treatment with TMZ. We also verified if these compounds were able to induce oxidative stress, cell cycle arrest, and apoptosis in GBM cells. Finally, the intracellular molecular mechanisms of lichen secondary metabolites and TMZ were further analyzed using microarrays in a 3D spheroid model of GBM.

## 2. Materials and Methods

### 2.1. Reagents and Standards

Eagle’s minimum essential medium (EMEM), Dulbecco’s modified Eagle medium (DMEM), and all other media components, including antibiotics solution (10,000 units penicillin and 10 mg streptomycin/mL), glutamine, nonessential amino acids (NEAA), and sodium pyruvate (NaP), were obtained from Merck (Darmstadt, Germany). Fetal bovine serum (FBS) was obtained from Biowest (Nuaillé, France). Other compounds used in the study, e.g., dimethylsulfoxide (DMSO), ribonuclease A (RNase A), 3-(4,5-dimethylthiazol-2-yl)-2,5-diphenyltetrazolium bromide (MTT), trypsin, Tris, PKF118–310, temozolomide (TMZ), and others were purchased from Merck (Darmstadt, Germany).

### 2.2. Lichens Collection, Extracts Preparation, and Lichen Secondary Metabolites Isolation

Manual collection of the examined lichens was performed: *Cladonia uncialis*, *Hypocenomyce scalaris*, and *Hypogymnia physodes* were collected from Jastrzębsko Stare ((52°17′56″ N 16°03′37″ E), Greater Poland, Poland (June 2018); *Parmelia sulcata* was found in Podlesice (50°33′49″ N 19°32′02″ E), Silesian region, Poland (July 2018); whereas *Platismatia glauca* originated from the forest belonging to the Piła commune (53°8′40.13″ N; 16°41′50.46″ E), Greater Poland, Poland (October 2018). The lichens were authenticated by Dr. Daria Zarabska-Bożejewicz from the Institute for Agricultural and Forest Environment of Polish Academy of Sciences in Poznan and Dr. Wojciech Gruszka from the Department of Biological Sciences, Poznan University of Physical Education. A voucher specimen (CUES 2018.06; HSES 2018.06; HPES 2018.06; PSES 2018.07, PGES 2018.10) was deposited in the herbarium of the Department of Pharmacognosy at Poznan University of Medical Sciences.

Next, the lichen-derived extracts were prepared by gradually extracting the dried, cleaned, and fragmented thallus (22.0 g for *P. glauca*, 12.0 g for *H. physodes*, 33.5 g for *C. uncialis*, 6.0 g for *P. sulcata*, and 1.5 g for *H. scalaris*). For *P. glauca*, the extraction with shaking at room temperature was effectuated. Hexane (eight times with 200 mL) (Avantor, Gliwice, Poland), followed by diethyl ether (five times with 200 mL) (Avantor, Gliwice, Poland), was used. *H. physodes* was extracted under the same experimental conditions but using hexane (five times with 200 mL) and then acetone (three times with 150 mL) (Avantor, Gliwice, Poland) as extractants. The thallus of *C. uncialis*, *P. sulcata*, and *H. scalaris* were extracted using an ultrasound bath at 35–40 °C. For *C. uncialis,* hexane (five times with 150 mL), followed by acetone (six times with 150 mL), was used. For *P. sulcata*, the hexane (three times with 80 mL) and acetone (fourteen times with 50 mL) were applied, and for *H. scalaris*, acetone extraction only was carried out (six times with 20 mL). Next, the extracts obtained with one kind of solvent were poured together and filtered using Whatman filter paper No. 1. A concentration under a vacuum at 35–40 °C (until dried) was effectuated next. The extracts were obtained with different yields: 0.8% for hexane and 4.9% for acetone extract from *P. glauca*, 0.3% for hexane and 6.5% for acetone extract from *H. physodes*, 0.9% for hexane and 0.8% for acetone extract from *C. uncialis*, 1.8% for hexane and 4.7% for acetone extract from *P. sulcata*, and 18.9% for acetone extract from *H. scalaris*.

Atranorin was purchased from ChromaDex (Los Angeles, CA, USA), but all other lichen secondary metabolites were isolated from the extracts as previously described [15]. Briefly, caperatic acid was isolated using the diethyl ether extract from *P. glauca* and the column chromatography (diameter 1.5 cm and length 8 cm of chromatographic column filling, silica gel 230–400 mesh, Sigma-Aldrich, St. Louis, MO, USA, with the gradient of hexane-ethyl acetate 100:0 to 60:40). The method was performed to obtain caperatic acid with a yield of 35%. Acetone extract from *H. physodes* was used to obtain the physodic acid. Thus, physodic acid was isolated by applying silica column chromatography (diameter 1.5 cm and length 8 cm of chromatographic column filling, silica gel 230–400 mesh, Sigma-Aldrich, St. Louis, MO, USA) using the increasing gradient mixtures of solvents toluene-ethyl acetate 110:0 to 100:10 as the mobile phase. The compound was obtained with a yield of 4%. Squamatic acid was obtained from the *C. uncialis* acetone extract by the spontaneous crystallization during evaporation of acetone with a yield of 33%. Salazinic acid was obtained by recrystallizing the *P. sulcata* acetone extract from the acetone: water (8:2) mixture with a yield of 42%. Lecanoric acid was isolated from the *H. scalaris* acetone extract, using preparative thin layer chromatography (PLC 60 F254, 2 mm, Merck, Darmstadt, Germany; solvent: diethyl ether-glacial acetic acid 100:1, ethyl acetate was utilized to wash the silica gel). The identity of the compounds was confirmed using UV, 1H NMR, 13C NMR, and MS analysis, and the obtained data were compared to the published values [16]. In addition, the purity of the isolated substances was estimated based on the chromatograms effectuated with high-performance liquid chromatography [14] or thin layer-chromatography with the standard solvent G (toluene-ethyl acetate-formic acid 99% (139:83:8)) or standard solvent C (toluene-glacial acetic acid (170:30)). Taking into account these analytical analyses, the purity of the tested compound was estimated as high (≥90%). The purity of the commercially purchased atranorin was declared as 97.1%. The compounds were dissolved in DMSO to obtain the stock solution of 20 mM, then they were aliquoted to reduce the freeze-thaw cycles, and stored at −20 °C.

### 2.3. Permeability through the Blood–Brain Barrier (PAMPA-BBB)

The parallel artificial membrane permeability assay (PAMPA) for the blood–brain barrier (BBB) (Pion Inc., Billerica, MA, USA) was used to analyze whether the lichen secondary metabolites tested in this study were able to cross the BBB. The stock solutions (5 mg/mL) of tested compounds were prepared using DMSO. Next, the donor solution (24.75 µg/mL) using Prisma buffer (pH = 7.4; Prisma HT, Pion Inc., Billerica, MA, USA) were made. Firstly, 180 µL of the donor solution were added to the donor wells. Then the filter membranes were coated with 5 μL BBB-1 lipid solution (Pion Inc., Billerica, MA, USA) and the acceptor wells were filled with 200 µL BSB (Brain Sink Buffer, Pion Inc., Billerica, MA, USA). The plates were sandwiched together and incubated for 4 h at 37 °C (Thermo Scientific MaxQ 4450, Waltham, MA, USA). The results were determined by spectroscopic analysis (Multiskan GO microplate reader, Waltham, MA, USA), with the appropriate wavelength for a given compound (240 nm for caperatic acid and salazinic acid, 250 nm for atranorin and squamatic acid, 263 nm for physodic acid, and 270 nm for lecanoric acid). The amount of permeated active compounds was calculated using the standard curves prepared for each tested compound. The theophylline and lidocaine were used as the negative and positive controls, respectively. Effective permeability (*P_e_*) of the compounds was calculated by using the following equation:

$$P_{e}=-\frac{ln\left(1-\frac{C_{A}}{C_{eq}}\right)}{S\times \left(\frac{1}{V_{D}}+\frac{1}{V_{A}}\right)\times t}$$

where: *P_e_* is the effective permeability coefficient (cm/s), *V_D_*—donor volume, *V_A_*—acceptor volume, *C_eq_*—equilibrium concentration,$\;C_{eq}=\frac{C_{D}\times V_{D}+C_{A}\times V_{A}}{V_{D}+V_{A}}$, *S*—membrane area, and *t*—incubation time (in seconds) [17].

Compounds with *P_e_* (×10^−6^ cm/s) > 1.5 were classified as high permeation predicted, while *P_e_* (×10^−6^ cm/s) < 1.5 were classified as low permeation predicted [17,18]. Samples were analyzed in triplicate, and the average was reported.

### 2.4. Two (2D) and Three Dimensional (3D) Cell Culture

Three human GBM cell lines were used in this study, i.e., A-172 (ATCC-CRL-1620), T98G (92090213), and U-138 MG (ATCC-HTB-16). The A-172 and U-138 MG cell lines were purchased from the American Type Culture Collection (ATCC), whereas the T98G cell line was obtained from the European Collection of Authenticated Cell Cultures (ECACC). It has been confirmed by us that O^6^-methylguanine-DNA methyltransferase (*MGMT*), the key factor determining the response to alkylating agents, such as TMZ, is expressed in T98G and U-138 MG cells, whereas in A-172 the expression of *MGMT* is silenced by promoter methylation [19]. Therefore, the cell lines differ in terms of TMZ sensitivity; A-172 is regarded as TMZ sensitive, whereas T98G and U-138 MG are TMZ resistant.

The cells were grown as monolayers (2D cell culture) in the recommended media: A-172 were grown in the ATCC-formulated DMEM (Merck, Darmstadt, Germany), whereas T98G and U-138 MG cell lines were grown in the ATCC-formulated EMEM (Merck, Darmstadt, Germany). These media were supplemented with FBS (Biowest, Nuaillé, France) to a final concentration of 10%, as well as antibiotics (penicillin and streptomycin) (Merck, Darmstadt, Germany) to the final concentrations of 1%. For the experiments, the amount of FBS was reduced to 5%. The medium for the T98G cell line was supplemented with 2 mM glutamine, 1% nonessential amino acids, and 1% sodium pyruvate (all purchased from Merck, Darmstadt, Germany).

Moreover, the 3D culture of the T98G cell line was also established. The cells were cultured in ultra-low attachment microplates (Corning^®^, Corning, NY, USA) (1000 cell per well) to form spheroids. The standard medium was supplemented with Corning^®^ Matrigel^®^ Matrix for Organoid Culture (3.5%) (Corning^®^, Corning, NY, USA) and epidermal growth factor—EGF (10 ng/mL) (Stemcell Technologies, Vancouver, BC, Canada). Both 2D and 3D cell cultures were grown in a standard humidified incubator (Memmert, Schwabach, Germany) at 37 °C in 5% CO_2_ and 95% air atmosphere.

### 2.5. MTT Test

In order to analyze the cytotoxicity of the analyzed lichen secondary metabolites, as well as to establish the appropriate concentrations for further analysis (allowing the survival of >70% of cells), we performed the MTT test. The procedure of the test was described previously [19]. Briefly, the cells (10,000 cells per well) were seeded on the 96-well plates and left for 24 h. Afterwards, the analyzed compounds in the concentration range of 1 µM up to 100 µM were added to the cells and left for 48 h incubation. DMSO treated cells served as control. After the above-mentioned time, the cells were washed twice with warm PBS and incubated for 3 h in the presence of a fresh medium containing MTT salt (0.5 mg/mL). Then the formazan crystals were dissolved in acidic isopropanol. The absorbance was measured at 570 and 690 nm on a Tecan Infinite M200 microplate reader (Grödig, Austria). The experiments were repeated at least three times.

### 2.6. Cell Culture Treatment

Based on the MTT test, the lichen secondary metabolite concentrations allowing the survival of more than 70% of cells were chosen for the subsequent experiments. The compounds were added as follows: A-172 cells—atranorin 25 µM, caperatic acid 50 µM, physodic acid 25 µM, squamatic acid 25 µM, salazinic acid 50 µM, and lecanoric acid 100 µM; T98G cells—atranorin 50 µM, caperatic acid 100 µM, physodic acid 25 µM, squamatic acid 50 µM, salazinic acid 100 µM, and lecanoric acid 100 µM; U-138 MG cells—atranorin 25 µM, caperatic acid 50 µM, physodic acid 25 µM, squamatic acid 50 µM, salazinic acid 50 µM, and lecanoric acid 100 µM. As far as the TMZ treatment is concerned, it was added in the concentrations depending on the *MGMT* expression and methylation status, i.e., TMZ 30 µM was added to the A-172 cell line, and TMZ 100 µM was added to both the T98G and U-138 MG cell lines. In the case of the 2D culture, the cells were treated with the compounds 24 h after seeding, whereas, in regard to 3D culture, 5 days were needed to form spheroids. All subsequent analyses were performed 48 h after treatment.

### 2.7. RNA Isolation, cDNA Synthesis and qPCR

The Universal RNA Purification Kit (EURx, Gdańsk, Poland) was used for total RNA isolation from the A-172, T98G, and U-138 MG cells, and the Revert-Aid First Strand cDNA Synthesis kit (Fermentas, Burlington, ON, Canada) was chosen for cDNA synthesis. Quantitative real-time PCR (qPCR) was performed using Hot FIREPol EvaGreen qPCR Mix Plus (Solis Biodyne, Tartu, Estonia) and LightCycler 96 (Roche Diagnostics GmbH, Mannheim, Germany). Primer sequences were previously published [19,20] and their synthesis was performed at the Institute of Biochemistry and Biophysics, Warsaw, Poland. The protocol started with 15 min enzyme activation at 95 °C, followed by 40 cycles of 95 °C for 15 s; appropriate annealing temperature for 20 s; 72 °C for 40 s and the final elongation at 72 °C for 5 min. Melting curve analysis was used to confirm the generation of a single amplification product. TATA box-binding protein (*TBP*), a component of the DNA-binding protein complex TFIID, served as the endogenous RNA control. The results were expressed as N-fold differences in the gene expression relative to the *TBP* gene. The ΔΔCt method was used for fold-change calculation. All samples were analyzed in triplicate, and the reaction was repeated at least twice.

### 2.8. Reactive Oxygen Species Generation Analysis

The intracellular detection of superoxide radicals was detected by flow cytometric analysis after the staining with dihydroethidium, using The Muse^®^ Oxidative Stress Kit (Merck, Darmstadt, Germany). Briefly, 100,000 cells per well were seeded on 6-well plates and after a 24 h incubation they were treated with the analyzed lichen secondary metabolites in the concentrations allowing >70% of cell survival; cells treated with DMSO were used as the negative control. After 48 h incubation, the cells were trypsinized, washed with PBS buffer, and resuspended in assay buffer containing Working Solution of Muse^®^ Oxidative Stress Reagent. The cells were incubated at 37 °C for 30 min, and then run on Muse^®^ Cell Analyzer (Merck, Darmstadt, Germany). The obtained data were evaluated using Muse^®^ Analysis Software ver. 1.5 (Merck, Darmstadt, Germany).

### 2.9. Cell Cycle Distribution Analysis

The Muse Cell Cycle Kit and the Muse Cell Cycle Software (both Merck, Darmstadt, Germany) were used to quantitatively measure the percentage of cells in the G0/G1, S, and G2/M phases of the cell cycle. The assay utilizes the nuclear DNA intercalating stain propidium iodide and RNAse A in a formulation. Propiodium iodide discriminates cells at different stages of the cell cycle, based on differential DNA content, while the presence of RNAse increases the specificity of DNA staining. Briefly, the cells were seeded on 6-well plates (100,000 cells per well) and incubated for 24 h. Afterward, the lichen secondary metabolites were added to the concentrations chosen based on the MTT test, allowing >70% of cell survival. 0.5% DMSO and 100 nM topotecan were used as negative and positive controls, respectively. The analysis was performed after a 48 h incubation with lichen secondary metabolites and started with washing the cells with PBS, followed by cell fixation with ice-cold ethanol and overnight incubation at −20 °C. Following steps included cell re-washing with PBS, then a 30 min incubation in the dark with propiodium iodide-RNAse A formulation, and a final analysis on Muse™ Cell Analyzer (Merck, Darmstadt, Germany).

### 2.10. Apoptosis Analysis

The fluorescent-based analysis of apoptosis using Muse^®^ Caspase-3/7 Kit was performed in order to analyze the percentage of live, early, and late apoptotic; total apoptotic; and dead cells in A-172, T98G, and U-138 MG GBM cells treated with lichen secondary metabolites. The principle of the method relies on the measurement of the activity of executioner caspases, i.e., caspase-3 and caspase-7, as well as analyzing the integrity of the cell membrane with the use of a dead cell marker 7-AAD. Cell seeding and treatment followed the same protocol as for cell cycle or ROS generation analysis, while the subsequent procedures were performed according to the manufacturer’s recommendations.

### 2.11. Immunohistochemistry Analysis

After 5 days, the spheroids were fixed in 4% phosphate-buffered formalin, embedded in paraffin, and sectioned. Sections were stained with hematoxylin and eosin (H&E), according to routinely used Department of Histology and Embryology protocols, to visualize cellular content. They were subsequently analyzed under a light microscope.

### 2.12. Microarray Expression Studies

The total RNA from lichen secondary metabolites and TMZ supplemented spheroids culture was isolated with the RNeasy MinElute, according to the manufacturer’s instructions (Qiagen, Hilden, Germany). The RNA for transcriptome study was received from three independent replicates for each experimental variant: control (1), TMZ 100 µM (2), atranorin 25 µM + TMZ 100 µM (3), caperatic acid 50 µM + TMZ 100 µM (4), physodic acid 25 µM + TMZ 100 µM (5), squamatic acid 25 µM + TMZ 100 µM (6), salazinic acid 50 µM + TMZ 100 µM (7), and lecanoric acid 100 µM + TMZ 100 µM (8), and then pooled together into a single sample per group.

The complete procedure for preparing RNA for hybridization was performed using the GeneChip WT PLUS Reagent Kit (Affymetrix, Santa Clara, CA, USA). The detailed procedure was described earlier [21]. First, a two-step cDNA synthesis reaction from 100 ng RNA using random primers extended by the T7 RNA polymerase promoter sequence was carried out. CRNA was synthesized by performing in vitro transcription for 16 h at 40 °C. Next, the cRNA was purified and re-transcribed into cDNA. CDNA was then biotin labeled and fragmented using the Affymetrix GeneChip WT Terminal Labeling and Hybridization kit (Affymetrix, Santa Clara, CA, USA). The biotin-labeled fragments of cDNA were hybridized by the Affymetrix Human Gene 2.1 ST ArrayStrip (20 h, 48 °C). Then, the microarrays were stained using the Affymetrix GeneAtlas Fluidics Station (Affymetrix, Santa Clara, CA, USA). The array strips were scanned using an Imaging Station of GeneAtlas System (Thermo Fisher Scientific, Waltham, MA, USA). The preliminary analysis of the scanned chips was performed with the use of Affymetrix GeneAtlas Operating Software (Affymetrix, Santa Clara, CA, USA). The quality of gene expression data was confirmed using the software’s quality control criteria.

### 2.13. Microarray Data Analysis

The obtained CEL files were further analyzed using the R statistical language and Bioconductor package, including the relevant Bioconductor libraries. For the normalization, background correction, and calculation of the expression values of the examined genes, the robust multiarray average (RMA) normalization algorithm implemented in the “Affy” library was applied [22]. A complete gene data table involving normalized gene expression values, gene symbols, gene names, and Entrez IDs was generated on the basis of assigned biological annotations taken from the “pd.hugene.2.1.st” library. The established cut-off criteria for differentially expressed genes (DEGs) were based on the differences in the absolute value from the expression fold change (FC) greater than 2. Genes fulfilling the aforementioned selection criteria were subjected to functional annotation and clusterization using the DAVID (Database for Annotation, Visualization, and Integrated Discovery) bioinformatics tool [23]. Gene IDs of differentially expressed genes were uploaded to DAVID by the “RDAVIDWebService” BioConductor library [24], where DEGs were assigned to relevant GO terms, with the subsequent selection of significantly enriched GO terms from GO BP DIRECT database. The *p*-values of selected GO terms were corrected using Benjamini-Hochberg correction. In similar way, the “RDAVIDWebService” library was carried out to analyze enrichment of the KEGG (Kyoto Encyclopedia of Genes and Genomes) signaling pathway database. The associations of individual DEGs with relevant KEGG signaling pathways were presented using Cytoscape (v. 3.7.2) software [25].

### 2.14. Statistical Analysis

The statistical significance between the experimental groups and their respective controls (in cytotoxicity tests, qPCR, and flow cytometry analysis) was assessed using Student’s t test, while ANOVA with Duncan’s multiple range post hoc test (Statistica 13.3, StatSoft Poland, Kraków, Poland) variance analysis with *p* < 0.05 considered as significant was applied for the PAMPA-BBB assay.

## 3. Results

### 3.1. Lichen Secondary Metabolites Cross the Blood-Brain Barrier (BBB)

The PAMPA-BBB assay revealed that all analyzed lichen secondary metabolites, except squamatic acid and salazinic acid, can cross the BBB (Table 1). In this regard, caperatic acid and physodic acid were characterized by the highest P_e_ coefficient, followed by atranorin and lecanoric acid.

### 3.2. Lichen Secondary Metabolites Reduce the Viability of GBM Cells in a Dose-Dependent Manner

**Figure 1:**
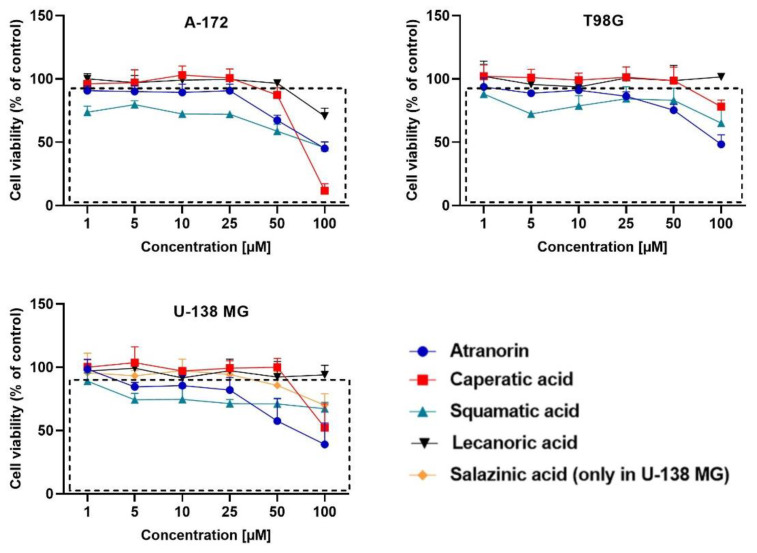
The effect of atranorin, caperatic acid, squamatic acid, and lecanoric acid on the viability of A-172, T98G and U-138 MG cells. In case of U-138 MG cells, the results for salazinic acid are also presented (the results for A-172 and T98G cells were previously published [5]). Statistically significant differences in cell viability between the cells exposed to the analyzed lichen secondary metabolites as compared to the control cells is indicated with a rectangle. The mean values ± SEM from three independent experiments are presented.

Here, as shown in Figure 1, the effect of atranorin, caperatic acid, squamatic acid and lecanoric acid on GBM cells viability is presented. For the results of the MTT test of physodic acid and salazinic acid (A-172 and T98G), as well as PKF118-310, we refer to our previously published papers [5,14,19], respectively. The results indicate that the effects of the 48 h treatment with lichen secondary metabolites were dose-dependent, relatively similar in all three GBM cell lines analyzed, and they did not depend on the *MGMT* methylation status nor the sensitivity to TMZ. Among the four compounds analyzed in this study, the most cytotoxic were squamatic acid and atranorin, which reduced the number of living cells in the whole analyzed concentration range, i.e., 1–100 µM (however, in T98G and U-138 MG cells the lowest tested concentration, i.e., 1 µM, was not cytotoxic). Importantly, caperatic acid in the highest tested concentration also had a significant impact on GBM cell viability. In the A-172 cell line, it reduced the viability of cells to only 11.67 ± 5.51%. Lecanoric acid was, on the other hand, not cytotoxic; only the A-172 cell line was sensitive to its maximal tested concentration (100 µM), which killed ~30% of cells. Referring to the previously published results of physodic acid, its cytotoxicity was remarkable, and the U-138 MG cell line was the most prone to its cytotoxic influence [14]. Salazinic acid was only toxic in the highest tested concentration in the A-172 and T98G cell lines [5], while in U-138 MG the 50 µM concentration also significantly reduced the number of viable cells. The IC_50_ values of the analyzed compounds together with their chemical structures are presented in Table 2. 

### 3.3. Physodic Acid and Squamatic Acid Induce Oxidative Stress in the T98G Cell Line

**Figure 2:**
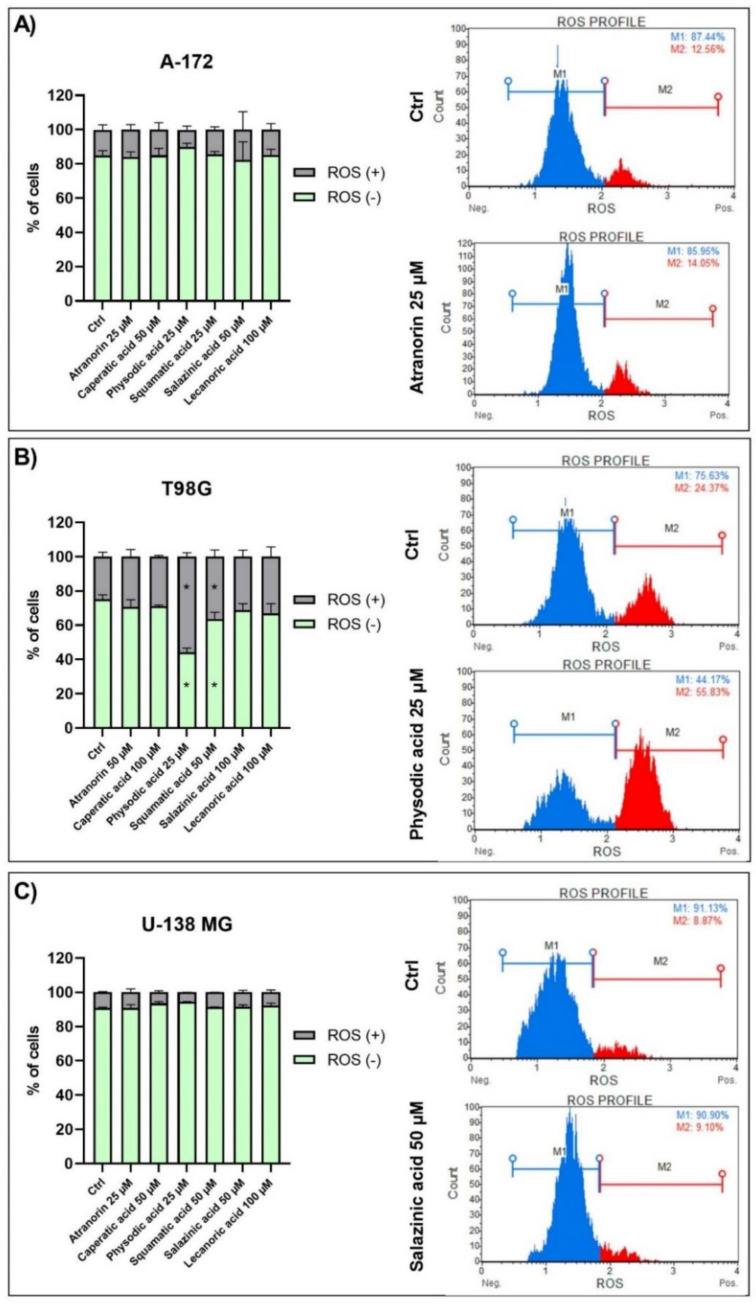
The impact of lichen secondary metabolites on ROS (superoxide) generation in the A-172 (panel (**A**)), T98G (panel (**B**)), and U-138 MG (panel (**C**)) cell lines after 48 h of treatment. ROS (+) denotes cells exhibiting ROS, while ROS (−) denotes the live cells not exhibiting ROS. Cells treated with vehicle (DMSO) are designated as Ctrl. Exemplary histograms are presented showing the results of DMSO (Ctrl) and one chosen compound. Values are shown as mean ± SEM calculated from three independent experiments. (*) indicates statistically significant differences from control group, *p* < 0.05.

One of the most important features of anticancer agents is the ability to generate oxidative stress. The results of our study show that physodic and squamatic acids induce oxidative stress in the T98G cell line (Figure 2). However, the level of superoxide radical was not elevated in the two other analyzed cell lines following lichen secondary metabolites treatment.

### 3.4. Lichen Secondary Metabolites Inhibit the Wnt/β-Catenin Pathway Target Genes in GBM Cell Lines, Regardless of Their MGMT Status and TMZ Resistance

**Figure 3:**
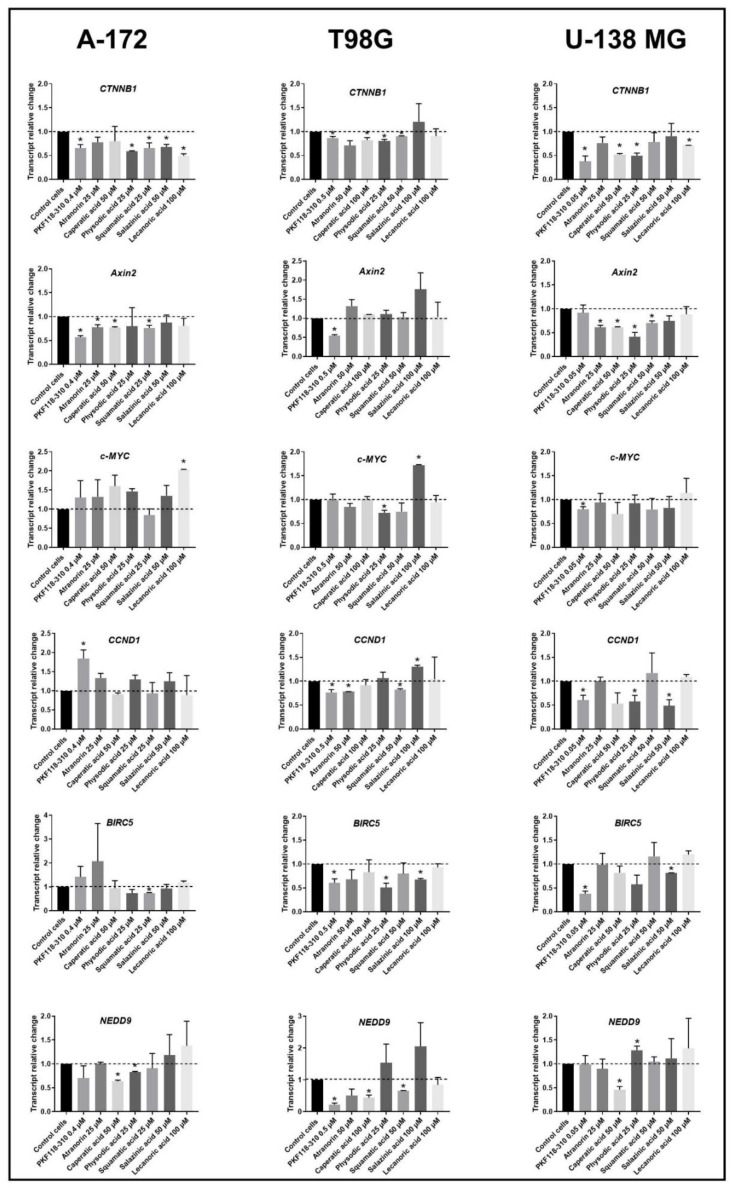
The effect of lichen secondary metabolite treatment on the expression of β-catenin (*CTNNB1*) and its target genes after 48 h of treatment of A-172 (left column), T98G (middle column), and U-138 MG (right column) cell lines. Means ± SEM from at least two separate experiments with three replicates in each are presented. The values were calculated as mRNA level in comparison with control cells treated with DMSO (expression equals 1). The asterisk (*) above the bar denotes a statistically significant difference from the control cells, *p* < 0.05.

Since Wnt/β-catenin signaling is regarded as a therapeutic target for GBM [11], we decided to verify whether lichen secondary metabolites do interfere with this critical cellular pathway and, if so, whether this influence is beneficial in terms of GBM cell growth inhibition. In order to achieve that, we analyzed the expression of the key molecule of the pathway, β-catenin, as well as the Wnt pathway target genes, namely *Axin2*, *c-MYC*, *CCND1*, *BIRC5,* and *NEDD9*. The results shown in Figure 3 provide evidence that the expression of β-catenin is downregulated in all three cell lines after the treatment with most of the analyzed lichen secondary metabolites. The results obtained are similar to those observed for PKF118-310, the Wnt pathway inhibitor. Regarding *Axin2*, its expression was mostly affected in the A-172 and U-138 MG cell lines. In this context, atranorin, caperatic, and squamatic acids downregulated its expression in both cell lines; in U-138 MG physodic acid also decreased *Axin2* expression. As far as *c-MYC* expression is concerned, we observed either no impact of lichen secondary metabolites on its mRNA level, or rather the upregulation; in this context lecanoric acid upregulated its expression in A-172 cell line, while salazinic acid did so in T98G cell line. In the latter cell line, only physodic acid downregulated *c-MYC* expression, while for atranorin and squamatic acid we observed a trend towards downregulation, but with no statistical significance. However, it has to be noted that in both A-172 and T98G cell lines even PKF118-310 did not diminish the level of *c-MYC* oncogene. Furthermore, we also analyzed *CCND1* expression, as this master cell cycle regulator is also one of the Wnt pathway target genes. Here the most profound effects were observed in the U-138 MG cell line, where physodic and salazinic acids downregulated *CCND1* expression by around half. Additionally, atranorin and squamatic acid exhibited similar downregulatory effects in the T98G cell line. Importantly, salazinic acid significantly upregulated *CCND1* expression in the latter cell line. The next gene analyzed was *BIRC5*, encoding survivin, the key apoptosis inhibitor. Here the most profound effects were observed in the T98G cell line, where physodic and salazinic acids downregulated *BIRC5* expression, similarly to what was observed for PKF118-310. Moreover, squamatic and salazinic acids diminished its expression in the A-172 and U-138 MG cell lines, respectively. Finally, *NEDD9* was also analyzed, as it regulates signaling complexes important in cell attachment, migration, and invasion as well as apoptosis and the cell cycle. Our study shows, that caperatic acid downregulated *NEDD9* expression in all analyzed cell lines. On the other hand, the effects of physodic acid treatment in the context of *NEDD9* expression were cell line dependent; in the A-172 cell line it downregulated, while in U-138 MG it upregulated its transcript level. Moreover, we also observed the downregulation of *NEDD9* after the treatment with squamatic acid in T98G cells.

### 3.5. Lichen Secondary Metabolites Affect Cell Cycle Distribution and Induce Apoptosis in the T98G Cell Line

**Figure 4:**
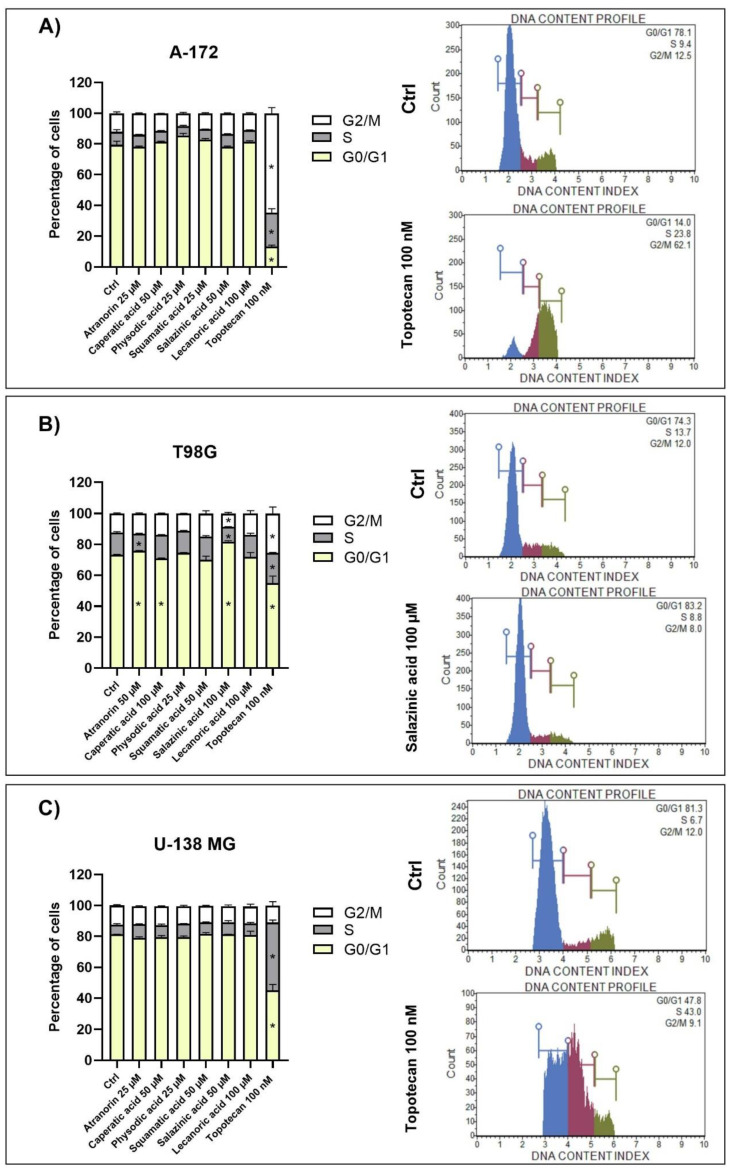
The effect of atranorin, caperatic acid, physodic acid, squamatic acid, salazinic acid, and lecanoric acid on the cell cycle distribution of A-172 (panel (**A**)), T98G (panel (**B**)), and U-138 MG cells (panel (**C**)). The percentage of cells in the G1/G0, S, and G2/M phases was analyzed by flow cytometry after staining with propidium iodide and RNase A. Topotecan was used as a positive control in this assay. Statistically significant differences (*p* < 0.05) in cell cycle distribution between the cells exposed to the analyzed lichen secondary metabolites as compared to the control cells treated with DMSO (Ctrl) are indicated with an asterisk (*). Representative histograms are shown on the right-hand side. The mean values ± SEM from three independent experiments are presented.

**Figure 5:**
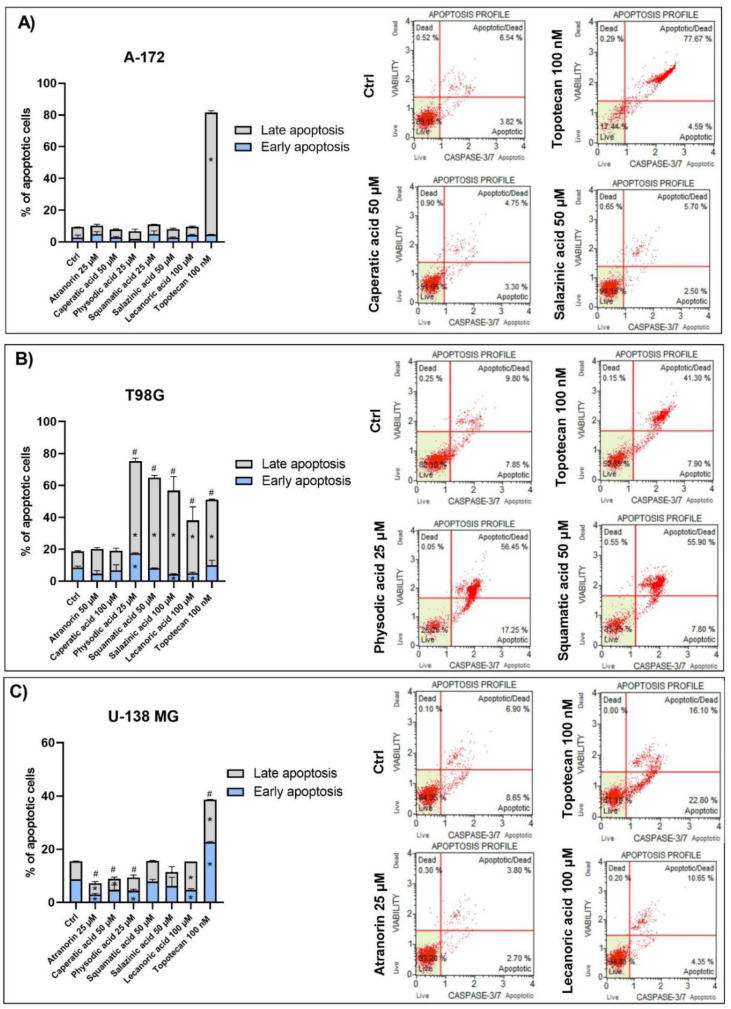
The effect of atranorin, caperatic acid, physodic acid, squamatic acid, salazinic acid, and lecanoric acid on the apoptosis of A-172 (panel (**A**)), T98G (panel (**B**)), and U-138 MG cells (panel (**C**)). The percentage of early and late apoptotic cells was analyzed by flow cytometry by the detection of caspase-3/7 activity and the integrity of the cell membrane with the use of a dead cell marker 7-AAD. Topotecan was used as a positive control in this assay. Statistically significant differences (*p* < 0.05) between the cells exposed to the analyzed lichen secondary metabolites as compared to the control cells treated with DMSO (Ctrl) are indicated with: (*) for early/late apoptotic cells and (#) for total apoptotic cells. Representative histograms are shown on the right-hand side. The mean values ± SEM from three independent experiments are presented.

Reduced cell viability can also be mediated by the cell cycle arrest and apoptosis induction. Therefore, we investigated these two phenomena using flow cytometry analysis after 48 h of treatment (Figure 4 and Figure 5, respectively).

The observed results were cell line-dependent, and T98G cells were found to be the most sensitive to lichen secondary metabolites treatment. The results and representative histograms are presented in Figure 4 and Figure 5 for cell cycle distribution and apoptosis analysis, respectively. Thus, in the T98G cell line, atranorin and salazinic acid slightly increased the number of cells in the G0/G1 phases and reduced the number of cells in the S phase of the cell cycle. Additionally, salazinic acid diminished the percentage of cells in the G2/M phases, and caperatic acid diminished the percentage of cells in the G0/G1 phases. Contrarily, we did not observe any statistically significant changes in cell cycle distribution in A-172 and U-138 MG cells.

As far as the pro-apoptotic effects of lichen secondary metabolites are concerned, such effects were detected in the T98G cell line. A significant increase in the number of apoptotic cells was observed after the treatment with physodic acid, squamatic acid, salazinic acid, and lecanoric acid. Similar results were obtained after the treatment with topotecan, which served as a positive control. Surprisingly, the opposite, i.e., an anti-apoptotic effect, was detected in the U-138 MG cell line after the treatment with atranorin, caperatic, and physodic acids. On the other hand, lecanoric acid decreased the percentage of early apoptotic cells, but increased the number of late apoptotic cells in the U-138 MG cell line.

### 3.6. Wnt/β-Catenin Pathway Inhibition Is Even Stronger When Lichen Secondary Metabolites Are Combined with TMZ

**Figure 6:**
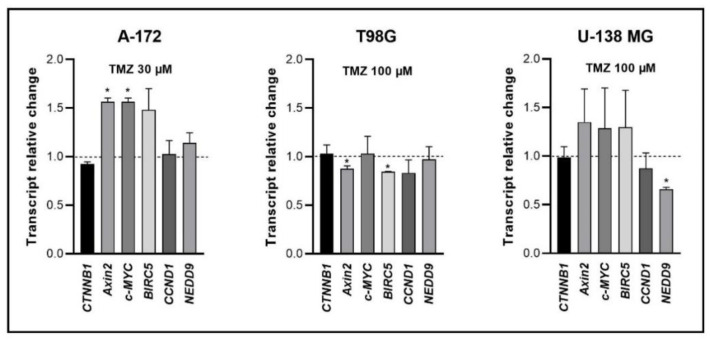
The effect of TMZ treatment on the expression of β-catenin (*CTNNB1*) and its target genes after 48 h of treatment of A-172, T98G, and U-138 MG cell lines. Means ± SEM from at least two separate experiments with three replicates in each are presented. The values were calculated as mRNA level in comparison with control cells treated with DMSO (expression equals 1). The asterisk (*) above the bar denotes a statistically significant difference from the control group, *p* < 0.05.

**Figure 7:**
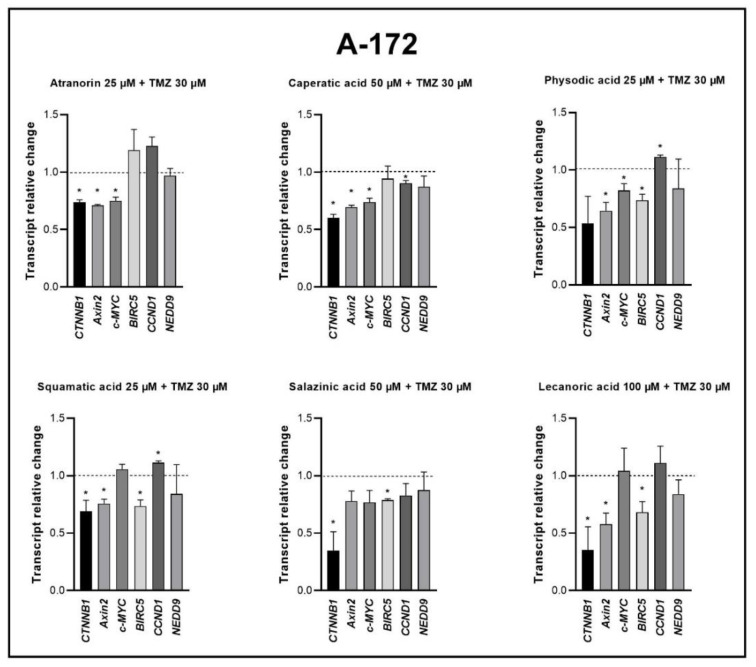
The effect of lichen secondary metabolites and TMZ treatment on the expression of β-catenin (*CTNNB1*) and its target genes after 48 h of treatment of the A-172 cell lines. Means ± SEM from at least two separate experiments with three replicates in each are presented. The values were calculated as mRNA levels in comparison with control cells treated with DMSO (expression equals 1). The asterisk (*) above the bar denotes a statistically significant difference from the control group, *p* < 0.05.

**Figure 8:**
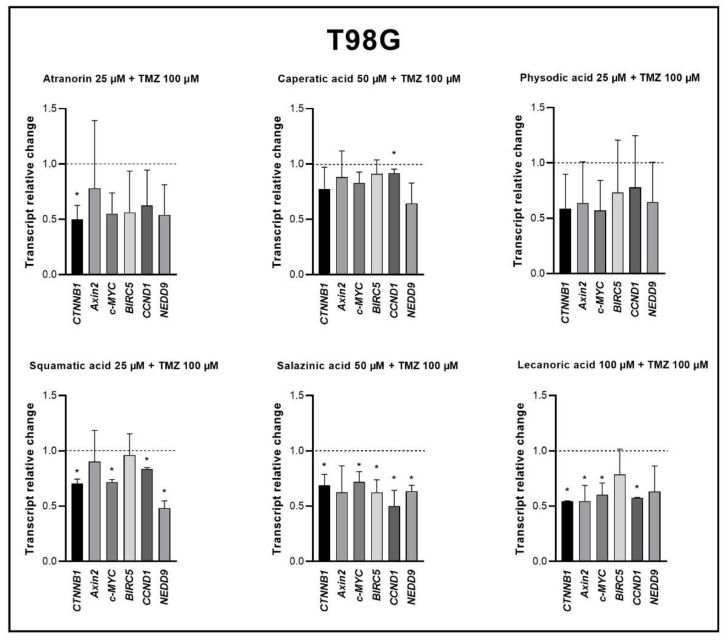
The effect of lichen secondary metabolites and TMZ treatment on the expression of β-catenin (*CTNNB1*) and its target genes after 48 h of treatment of the T98G cell lines. Means ± SEM from at least two separate experiments with three replicates in each are presented. The values were calculated as mRNA levels in comparison with control cells treated with DMSO (expression equals 1). The asterisk (*) above the bar denotes a statistically significant difference from the control group, *p* < 0.05.

**Figure 9:**
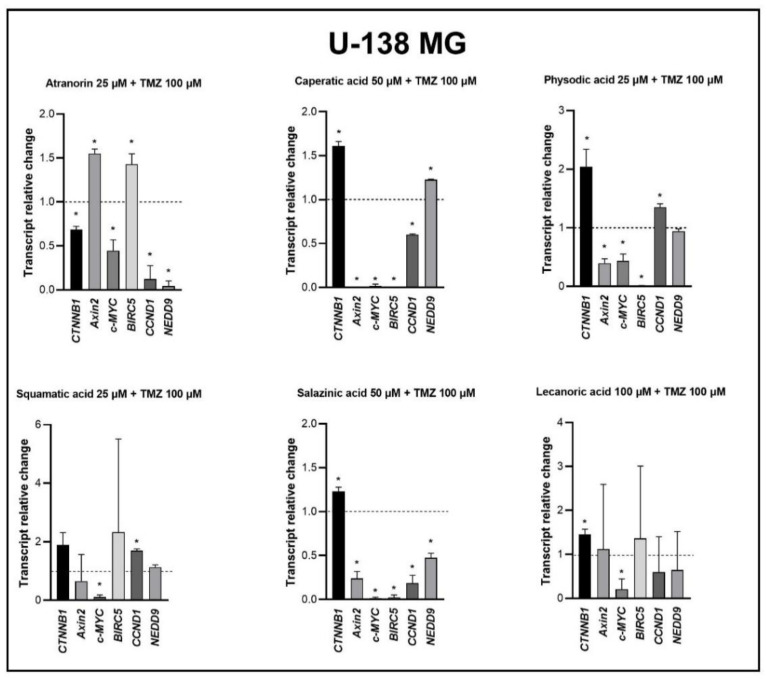
The effect of lichen secondary metabolites and TMZ treatment on the expression of β-catenin (*CTNNB1*) and its target genes after 48 h of treatment of the U-138 MG cell lines. Means ± SEM from at least two separate experiments with three replicates in each are presented. The values were calculated as mRNA levels in comparison with control cells treated with DMSO (expression equals 1). The asterisk (*) above the bar denotes a statistically significant difference from the control group, *p* < 0.05.

TMZ is a gold standard in GBM treatment, which is why we decided to verify whether combining lichen secondary metabolites with this alkylating drug would result in beneficial effects in terms of Wnt pathway inhibition. Thus, first we analyzed the impact of only TMZ (Figure 6) and then the combinations (Figure 7, Figure 8 and Figure 9, for A-172, T98G, and U-138 MG cells, respectively). Here we show that the treatment with TMZ upregulated the expression of *Axin2* and *c-MYC* in the TMZ sensitive A-172 cell line. Contrarily, in the TMZ resistant T98G cells it led to the downregulation of *Axin2* and *BIRC5*. Moreover, *NEDD9* was downregulated in U-138 MG cells after the treatment with 100 µM TMZ.

The analysis performed on the A-172 cell line revealed the downregulation, or a trend towards downregulation, of β-catenin and the genes controlled by the β-catenin/TCF/LEF transcriptional complex as a response to the treatment with lichen secondary metabolites and TMZ. Mostly, the mRNA level of *CTNNB1*, *Axin2*, *c-MYC*, and *BIRC5* was found to be diminished. All combinations downregulated from two to four out of six analyzed genes. Importantly, in case of physodic acid and squamatic acid combinations with TMZ, the upregulation of the cyclin D1 encoding gene (*CCND1*) was also observed. Detailed results are presented in Figure 7.

As far as the T98G cell line is concerned, the best results were obtained for salazinic acid, which downregulated five out of six analyzed genes (although a trend toward downregulation of the sixth gene, namely *Axin2*, was also observed). Also, squamatic acid and lecanoric acid treatment with TMZ led to the decrease of Wnt pathway target genes expression. In the case of squamatic acid, the downregulated genes were as follows: *CTNNB1*, *c-MYC*, *CCND1*, and *NEDD9*; while concerning lecanoric acid, these were *CTNNB1*, *Axin2*, *c-MYC*, and *CCND1*. Atranorin and TMZ decreased the expression of only β-catenin, caperatic acid and TMZ reduced the expression of *CCND1*, while physodic acid and TMZ did not downregulate any genes.

The treatment with lichen secondary metabolites and TMZ led to the most remarkable Wnt pathway inhibition in U-138 MG cells (Figure 9). In this cell line, the expression of the Wnt target genes was drastically diminished after the treatment with most lichen secondary metabolites and TMZ, even though the expression of β-catenin was upregulated in most cases (only atranorin and TMZ downregulated its mRNA level). Such a huge downregulatory effect was observed in respect to all the analyzed combinations, in particular in the cases of caperatic acid, physodic acid, and salazinic acid co-treatments with TMZ.

### 3.7. The Combination of Caperatic Acid and TMZ Results in the Most Altered Gene Expression Profile of T98G Cells—Derived Spheroids

**Figure 10:**
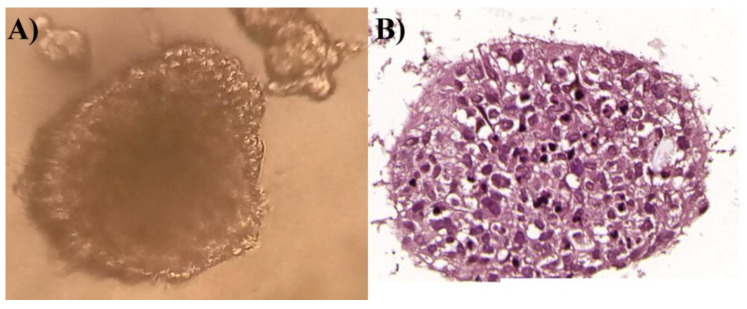
The T98G cell line has the ability to form spheroids in a suspension culture (panel (**A**)) which are characterized by well-defined shapes and edges. We also confirmed by H&E staining that those structures are composed of rapidly proliferating cells and no necrotic area can be distinguished (panel (**B**)).

In order to better understand the impact of lichen secondary metabolites on GBM cells, a 3D spheroid culture was generated using T98G cells—the cell line which was the most prone to oxidative damage, cell cycle changes, and apoptosis induction. Those 3D structures consisted of highly proliferative cells without the necrotic areas within them (Figure 10).

**Figure 11:**
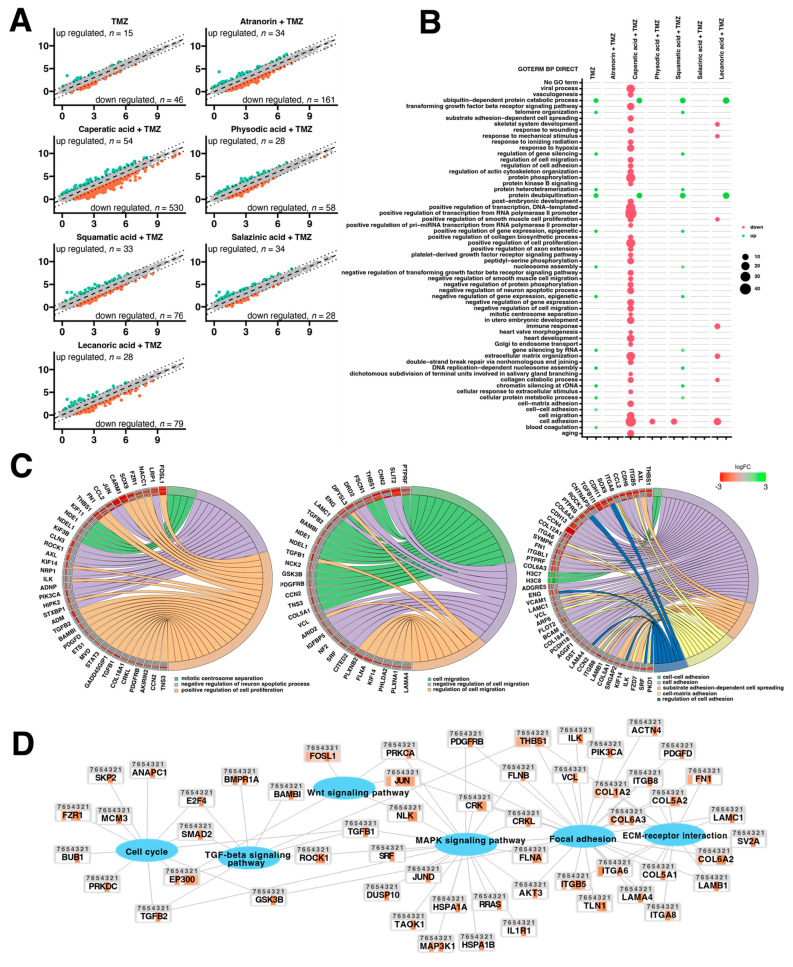
Gene expression profile based on microarray experiments. Variations in gene expression between the variants (specific lichen secondary metabolites with/or TMZ) vs. control are shown as scatter plots (**A**). The x and y values on the scatter plots are the normalized signal values, shown in a log2 scale. The black dotted lines were set as fold change cut-off value (FC) = 2 and −2. Bubble plot of differentially expressed genes from each group assigned to gene ontology (DAVID GO-BP DIRECT) terms where the red color represents GO terms whose genes are downregulated while green corresponds to GO terms of upregulated genes (**B**). Circos plots show the interdependence between selected GO terms and their genes. Symbols of DEGs are presented on the left side of the graph, where their fold-change values were mapped by color scale (green indicates higher expression, red indicates lower expression, and grey indicates expression levels below the cut-off value). This area was further subdivided into compartments corresponding to the regulation of the expression of the given gene in the relevant study groups. It represents the following comparisons from the outer side of the graph: TMZ vs. control, Atranorin + TMZ vs. control, Caperatic acid + TMZ vs. control, Physodic acid + TMZ vs. control, Squamatic acid + TMZ vs. control, Salazinic acid + TMZ vs. control, Lecanoric acid + TMZ vs. control (**C**). Kyoto Encyclopedia Genes and Genomes (KEGG) pathway analysis of differentially expressed genes. The significant pathways (e.g., Wnt signaling pathway) of regulated genes are demonstrated. Differentially expressed genes belonging to these pathways were assigned to a predetermined color scale which was subsequently imposed on the gene/protein symbol field. The whole area of the gene was divided into subareas marked with a number above, where: 1—TMZ vs. control, 2—Atranorin + TMZ vs. control, 3—Caperatic acid + TMZ vs. control, 4—Physodic acid + TMZ vs. control, 5—Squamatic acid + TMZ vs. control, 6—Salazinic acid + TMZ vs. control, 7—Lecanoric acid + TMZ vs. control (**D**).

The T98G spheroids were exposed to the influence of lichen secondary metabolites for 48 h and subjected to microarray analysis. A pairwise scatter plot analysis was performed to determine the general profiles of the whole gene expression in the control (1), TMZ 100 µM (2), atranorin 25 µM + TMZ 100 µM (3), caperatic acid 50 µM + TMZ 100 µM (4), physodic acid 25µM + TMZ 100 µM (5), squamatic acid 25 µM + TMZ 100 µM (6), salazinic acid 50 µM + TMZ 100 µM (7), and lecanoric acid 100 µM + TMZ 100 µM (8) experimental groups; this pairwise analysis revealed the patterns of upregulated and downregulated genes compared to the control (Figure 11A). Each color dot corresponds to one differentially-expressed transcript, with orange and green dots representing the downregulated and upregulated genes in each experimental group, respectively. A gene expression FC > abs. 2 and an adjusted *p* value of ≤0.05 were considered to indicate significantly changed gene expression. Based on those criteria, the number of significantly changed genes in all experimental groups vs. the control were, respectively, as follows: downregulated: 46 (TMZ 100 µM), 161 (atranorin 25 µM + TMZ 100 µM), 530 (caperatic acid 50 µM + TMZ 100 µM), 58 (physodic acid 25 µM + TMZ 100 µM), 76 (squamatic acid 25 µM + TMZ 100 µM), 28 (salazinic acid 50 µM + TMZ 100 µM), and 79 (lecanoric acid 100 µM + TMZ 100 µM) and upregulated: 15 (TMZ 100 µM), 34 (atranorin 25 µM + TMZ 100 µM), 54 caperatic acid 50 µM + TMZ 100 µM, 28 (physodic acid 25µM + TMZ 100 µM), 33 (squamatic acid 25 µM + TMZ 100 µM), 34 (salazinic acid 50 µM + TMZ 100 µM), and 28 (lecanoric acid 100 µM + TMZ 100 µM).

Differentially expressed genes from each group (TMZ 100 µM vs. control; atranorin 25 µM + TMZ 100 µM vs. control; caperatic acid 50 µM + TMZ 100 µM vs. control; physodic acid 25 µM + TMZ 100 µM vs. control; squamatic acid 25 µM + TMZ 100 µM vs. control; salazinic acid 50 µM + TMZ 100 µM vs. control; lecanoric acid 100 µM + TMZ 100 µM vs. control) were then assigned to Gene Ontology (GO). The GO analysis showed that the addition of lichen secondary metabolites and TMZ alters the expression of certain genes that play an essential role in the regulation of signaling pathways, inter alia: “positive regulation of cell proliferation”, “double−strand break repair via nonhomologous end joining”, extracellular matrix organization”, “cell migration”, “cell adhesion”, “protein deubiquitination”, “ubiquitin−dependent protein catabolic process”, “transforming growth factor beta receptor signaling pathway”, “nucleosome assembly”, and “regulation of actin cytoskeleton organization”. Note that the variant, caperatic acid 50 µM + TMZ 100 µM vs. control, resulted in the greatest number of altered biological processes (Figure 11B).

Due to the structure of the GO database, single genes can often be assigned to many ontological terms. For this reason, the relationship between genes and GO terms were mapped with circos plots, with visualization of logFC values and gene symbols. All of those genes were either upregulated (green color) or downregulated (red color) (Figure 11C) in the treated spheres compared to control. All these genes are involved in several different pathways activated after TMZ and lichen secondary metabolites treatment, including mitotic centrosome separation, cell migration, cell-cell adhesion, substrate adhesion-dependent cell spread, and cell-matrix adhesion.

A pathway analysis was also performed for the differentially expressed genes based on the Kyoto Encyclopedia Genes and Genomes (KEGG) database. This analysis allowed us to determine the biological pathways “Cell cycle”, “TGF-beta signaling pathway”, “Wnt signaling pathway”, “MAPK signaling pathway”, “Focal adhesion”, and “Focal adhesion”, which involve a significant enrichment of differentially expressed genes in the examined groups (*p* < 0.05). Differentially expressed genes belonging to these pathways were assigned to a predetermined color scale, which was subsequently imposed on the gene/protein symbol field (Figure 11D).

## 4. Discussion

Lichen secondary metabolites hold great promise for biopharmaceutical applications [1]. However, data regarding their potential use as anti-GBM agents are scarce. Novel drugs or drug combinations are urgently needed to prolong GBM patients’ lifespan, which is only around 14 to 20 months [26]. Currently, this disease is incurable and even though some clinical benefit is manifested after the gold standard therapy consisting of surgery, radiotherapy and TMZ chemotherapy (Stupp protocol), tumor relapse is almost inevitable [27]. Moreover, around 50% of patients do not benefit from TMZ, due to the MGMT-dependent resistance mechanism [26]. Another important factor contributing to the GBM chemo- and radiotherapy resistance is the hyperactivated Wnt/β-catenin pathway [11,28]. Thus, this signaling pathway has become the novel drug target for GBM treatment [11,12,29].

Therefore, in this study we examined the anti-GBM effects as well as the underlying molecular mechanisms, including the impact on Wnt/β-catenin and other cancer-related signaling pathways of lichen secondary metabolites used as a single compound or combined with TMZ. To the best of our knowledge, such a therapeutic approach has never been studied before. The metabolites derived from *H. scalaris* (lecanoric acid), *C. uncialis* (squamatic acid), *H. physodes* (physodic acid), *P. sulcata* (salazinic acid), and *P. glauca* (caperatic acid) were analyzed. We also analyzed atranorin, which is present in many lichen species [16]. Atranorin, lecanoric acid, and squamatic acid are depsides, physodic acid and salazinic acid are depsidones, and caperatic acid is a poly-carboxylic fatty acid. They were investigated using a 2D model of TMZ-sensitive (A-172) and TMZ-resistant (T98G and U-138 MG) GBM cell lines, supported by a 3D-spheroid model of T98G cells.

The initial obstacle that therapies against GBM must overcome is the BBB. Our results show that the permeability coefficient of all the analyzed compounds, except salazinic acid, was high enough to treat these compounds as BBB permeable. This opens up the pathway for their simple delivery to the CNS. In the case of salazinic acid, novel drug delivery systems, such as ligand-anchored dendrimers that utilize receptor-mediated transcytosis should be taken into consideration [11].

Our previous studies confirmed the cytotoxic potential of physodic acid and salazinic acid [5,14]. Here we show that atranorin, squamatic acid, and caperatic acid dose-dependently also reduce the number of living GBM cells. Additionally, lecanoric acid in the highest tested concentration, which was 100 µM, diminished the number of GBM cells. We assume that one of the mechanisms responsible for the cytotoxic potential of physodic acid and squamatic acid is oxidative stress generation, as was shown in our T98G cell line flow cytometry analysis. Another possible mechanism would be the interference with the cell cycle regulatory mechanisms. In our study, atranorin, caperatic acid, and salazinic acid altered the distribution of the cell cycle phases in the T98G cell line. In a study of Roser et al. 30 µg/mL atranorin and 30 µg/mL lecanoric acid significantly reduced the viability of HCT-116 cells; however, atranorin had no significant effect on the cell cycle distribution, whereas lecanoric acid caused HCT-116, NIH3T3, and HeLa cell cycle arrest in the G2 phase [30]. In another study, atranorin selectively inhibited MDA-MB-231 and MCF-7 breast cancer cells in a differential and dose-dependent manner with the IC_50_ concentration of 5.36  ±  0.85 μM and 7.55  ±  1.2 μM, respectively [31]. The cytotoxic activity of lichen secondary metabolites was also observed in different cancer cell line models, including lung cancer [32] or melanoma [33]. Importantly, the reports show higher cytotoxic properties of lichen-derived compounds to cancer cells as compared to non-cancer cells [34,35,36].

Our previous studies revealed that lichen secondary metabolites, especially caperatic acid and physodic acid, exert anticancer properties, inhibiting Wnt/β-catenin pathway in colorectal cancer cells [15,37]. Moreover, in a study by Zhou et al., atranorin was found to suppress β-catenin-mediated TOPFLASH activity by inhibiting the nuclear import of β-catenin and downregulating β-catenin/LEF and c-jun/AP-1 downstream target genes such as *CD44*, *CCND1*, and *c-MYC* in lung cancer cells [32]. To the best of our knowledge, our study is the first to show that in GBM cell lines, β-catenin, a major downstream effector of the Wnt pathway, is downregulated by lichen secondary metabolites, both when they are used as single agents or in the combination with TMZ. Additionally, the Wnt target genes were often downregulated, and the most significant downregulation was observed in U-138 MG cell line when lichen secondary metabolites, in particular caperatic acid, were combined with TMZ. Importantly, here we show that lichen secondary metabolites inhibit Wnt signaling even in the MGMT expressing, TMZ resistant GBM cell lines. This is in line with the study by Yun et al., who found that combinatorial therapeutic strategies of TMZ plus a small molecule inhibitor of the Wnt/β-catenin pathway act synergistically in GBM cells [28]. Taking into consideration that the Wnt/β-catenin signaling pathway induces TMZ-resistance [28], lichen secondary metabolites can be considered as a potential adjuvant therapy resensitizing cells to this chemotherapeutic drug.

Since Wnt signaling and apoptosis are interconnected, we assumed that lichen secondary metabolites might also induce apoptosis in GBM cells. This was found to be true in the case of the T98G cell line, but not in two other cell lines. Among all the compounds analyzed, physodic acid was found to induce the strongest pro-apoptotic effects, leading to almost 80% of apoptotic cells detected following 48 h treatment. Similar results were also obtained by Cardile et al., who found that physodic acid activated an apoptotic process, probably involving the reduction of Hsp70 expression, in A375 melanoma cancer cells [33]. In another study, the treatment of Jurkat cells with an extract from the lichen *Pseudevernia furfuracea* and its major constituent, physodic acid, resulted in intrinsic caspase-dependent cell death induction, which was associated with increased oxidative stress, DNA damage, and cell cycle arrest [38]. The authors report the activation of cell cycle checkpoint proteins p53, p21, and p27 and stress/survival kinases p38 MAPK, JNK, and PI3K/Akt as a result of *P. furfuracea* and physodic acid treatment [38]. In our study, squamatic acid, salazinic acid, and lecanoric acid also induced apoptosis in GBM cells, and the percentage of apoptotic cells was even higher as compared to the effects of the 100 nM anticancer drug, topotecan. These results are in line with a study by Nguyen et al., who found that among 17 lichens species, *Flavocetraria cucullata* exhibited the most potent cytotoxicity and pro-apoptotic effect in several human cancer cell lines and usnic acid, but also salazinic acid, squamatic acid, baeomycesic acid, d-protolichesterinic acid, and lichesterinic acid were found as major subcomponents of its acetone extracts [36]. Moreover, contrarily to our result, in a study of Roser et al. lecanoric acid did not induce apoptosis, whereas atranorin at 30 µg/mL significantly induced apoptosis in HCT-116 cells [30]. Harikrishnan et al. also reported that atranorin significantly downregulated the anti-apoptotic Akt, and increased the Bax level and caspases-3 activity in breast cancer cells [31]. These results clearly demonstrate that the pro-apoptotic effects of lichen secondary metabolites are cell line-dependent and require further study.

Additional lichenochemical target genes and signaling pathways were also revealed by our microarray analysis. Since spheroids mimic the in vivo behavior of cells better than monolayer culture, we decided to establish a 3D culture of the T98G cell line. This cell line was chosen for the spheroid formation for three reasons. First, because it was able to grow in a 3D culture; next, because it seemed the most prone to lichenochemical treatment, based on our ROS generation, cell cycle and apoptosis analysis; and finally, because it is TMZ resistant and exemplifies the most challenging tumor. Interestingly, our transcriptome profiling showed the most promising anti-GBM properties of the combination of caperatic acid and TMZ. Treatment with these two compounds resulted in the most significant downregulatory transcriptomic changes in cancer-related genes involved in e.g., vasculogenesis, TGF-β signaling, or positive regulation of proliferation, among others. Moreover, genes involved in cell adhesion, cell migration, and cell-matrix adhesion were found to be downregulated after the treatment with caperatic acid and TMZ. KEGG analysis also confirmed that all lichen secondary metabolites, but in particular caperatic acid and TMZ, interfere with the Wnt pathway, downregulating *FOSL1*, another of its target genes. Additionally, TGF-β and MAPK signaling pathways were identified as additional targets of lichen secondary metabolites combined with TMZ. This is an important finding, as TGF-β and MAPK pathways play critical roles in cell cycle regulation, as well as in tumor formation and metastasis [39]. It has been reported that MAPK signaling plays a key role in the co-activation of cell proliferation and CREB, a vital regulator of *CCND1* expression in GBM cells [40]. Indeed, the microarray as well as flow cytometry analysis confirmed that lichen secondary metabolites also interfere with the cell cycle. Additionally, the extracellular matrix (ECM)-receptor interaction pathway was also found to be targeted by the lichen secondary metabolites, especially caperatic acid and physodic acid when combined with TMZ. ECM-receptors pathways are implicated in the process of tumor shedding, adhesion, degradation, movement, and hyperplasia [41]. The interactions between the ECM and GBM microenvironment were also found to be crucial in tumor progression [42]. Further studies including the in vivo models are required in order to fully elucidate the molecular mechanisms exerted by lichen secondary metabolites, but our results suggest that these compounds may serve well as the adjuvants to the standard therapy of GBM patients.

## 5. Conclusions

We conclude that lichen secondary metabolites, in particular caperatic acid, but also atranorin, physodic acid, squamatic acid, salazinic acid, and lecanoric acid, ameliorate the response of GBM cells to TMZ treatment, in particular due to the Wnt pathway inhibition mechanism. We also showed that lichen secondary metabolites were able to generate oxidative stress, interfered with the cell cycle distribution, and induced apoptosis in the TMZ-resistant T98G cell line. The co-treatment of GBM cells with caperatic acid and TMZ resulted in the most significant and potentially therapeutic transcriptomic changes. Further studies are required to determine the potential clinical application of lichen secondary metabolites in GBM therapy. The safety of a co-treatment with TMZ for normal astrocytes and neurons should be evaluated and the best drug delivery system selected. Recognition of the subpopulation of GBM patients who would benefit from adjuvant lichen secondary metabolites therapy seems crucial. However, the good anti-GBM profile and BBB permeability of those compounds give hope for their successful implementation into treatment.

## Figures and Tables

**Table 1:** The effective permeability (*P_e_*) of lichen secondary metabolites using the parallel artificial membrane permeability assay for the blood-brain barrier (PAMPA-BBB).

Compounds	*P_e_* × 10^−6^ (cm/s)
Atranorin	2.2 ± 0.1
Caperatic acid	5.2 ± 0.1
Physodic acid	3.9 ± 0.2
Squamatic acid	0.5 ± 0.1
Salazinic acid	np
Lecanoric acid	1.7 ± 0.2
Theophylline	1.1 ± 0.2
Lidocaine	25.5 ± 2.6

np—classified as non-permeable (i.e., *P_e_* < 0.5 × 10^−6^ cm/s). Theophylline and lidocaine were used as negative and positive controls, respectively. The mean values ± SEM from three independent experiments are presented (*n* = 3). The statistical analysis of the *P_e_* values revealed that the tested metabolites differed significantly from one another in their ability to pass through the BBB; only atranorin, lecanoric acid, and theophylline belonged to the same statistical group, and thus, had similar BBB permeability.

**Table 2:** The chemical structure of the analyzed compounds and their IC_50_ values.

Compound	IC_50_ [uM] ± SEM			Chemical Structure
	A-172	T98G	U-138 MG	
Atranorin	90.89 ± 3.70	98.63 ± 7.80	47.84 ± 2.16	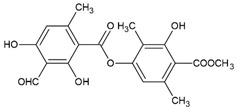
Caperatic acid	80.10 ± 1.88	>100	>100	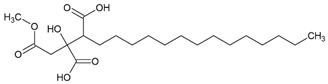
Physodic acid	42.41 ± 1.25 ^I^	50.57 ± 1.09 ^I^	45.72 ± 4.20 ^I^	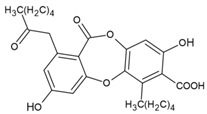
Squamatic acid	84.93 ± 3.62	>100	>100	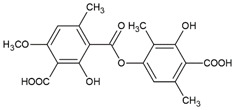
Salazinic acid	>100 ^II^	>100 ^II^	>100	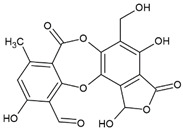
Lecanoric acid	>100	>100	>100	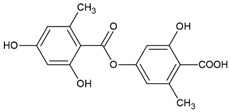

The IC_50_ is expressed as mean  ±  SEM obtained from three independent experiments and are calculated based on the dose–response curves assessed by the MTT assay or taken from the literature (I—[14], and II—[5]).

## Data Availability

Raw data files and a technical description were deposited in the Gene Expression Omnibus (GEO) repository at the National Center for Biotechnology Information (http://www.ncbi.nlm.nih.gov/geo/, accessed on 28 February 2022). The records have been assigned GEO accession number GSE199185.
